# Copines, a Family of Calcium Sensor Proteins and Their Role in Brain Function

**DOI:** 10.3390/biom14030255

**Published:** 2024-02-21

**Authors:** Mikhail Khvotchev, Mikhail Soloviev

**Affiliations:** 1 Department of Biochemistry, Center for Neuroscience, Faculty of Science, Mahidol University, Bangkok 10400, Thailand; 2 Department of Biological Sciences, Royal Holloway University of London, Egham, Surrey TW20 0EX, UK

**Keywords:** Copine protein, calcium signaling, calcium sensor, phospholipid binding, protein interactions, synapse, neurotransmitter release, synaptic plasticity, brain diseases

## Abstract

The Copines are a family of evolutionary conserved calcium-binding proteins found in most eukaryotic organisms from protists to humans. They share a unique architecture and contain tandem C2 domains and a Von Willebrand factor type A (VWA) domain. C2 domains in Copines bind calcium, phospholipids, and other proteins and mediate the transient association of these proteins with biological membranes at elevated calcium levels. The VWA domain also binds calcium and is involved in protein–protein interactions. Here, we provide a comprehensive review of the sequences, structures, expression, targeting, and function of the entire family of known Copine proteins (Copine 1–9 in mammals) with a particular emphasis on their functional roles in the mammalian brain. Neuronal Copines are implicated in a wide array of processes from cell differentiation to synaptic transmission and plasticity and are also linked to several pathological conditions from cancers to brain diseases. This review provides the most up-to-date insights into the structure and function of Copines, with an emphasis on their role in brain function.

## 1. Introduction

Copines were discovered simultaneously in ciliate Paramecium as abundant calcium-dependent phospholipid binding proteins [1] and in mammalian brains where expression of neuronal Copine 6 was upregulated by activity [2]. The protein name is derived from the French word for “companion” emphasizing the ability to associate with phospholipid membranes as one of the critical features of Copines [3]. Copines are found in many single-cell and multicellular eukaryotes from protists to humans. The number of Copine genes differs from one to two in protists to nine that exist in humans and other mammals (Copines 1–9) indicating a growing functional importance of Copine family in complex organisms. Interestingly, Copines are not present in yeast and Drosophila, suggesting that they emerged at a later stage of eukaryotic evolution and have been lost in some multicellular organisms.

Copines are implicated in multiple biological processes related to membrane dynamics, vesicle trafficking, intracellular signaling, cytoskeleton organization, neuronal development, cell adhesion, immune responses, and regulation of cell growth and apoptosis, however, it is not known whether they share a unified molecular mechanism of action. In round worm C. elegans, Copine controls synaptic expression of nicotinic acetylcholine receptors [4] and TRP channel GON-2 that stimulates gonadal cell division [5] and is required for myofilament stability [6]. In protist, social amoeba Dictiostellium, Copine A is important for cell differentiation, cytokinesis, contractile vacuole function, phospholipid distribution, and slug formation [7,8,9,10,11]. In Copine A mutants, slug formation deficits could be rescued by introducing a small number of wild-type cells into the amoeba population suggesting that Copine regulates the secretion of unknown factors essential for slug formation [12]. In plants, Copines regulate development, defense mechanisms, and response to low humidity conditions [13,14,15,16,17,18,19]. In mammalian cells, Copines are implicated in multiple signaling pathways. Copine 1 is required for calcium-dependent stimulation of the TNF-α signaling pathway [20], and it downregulates NF-κB [21]. Copine 3 regulates insulin secretion and glucose uptake in pancreatic β-cells [22]. Secreted Copine 7 regulates dental development and differentiation [23,24,25,26]. At the organismal level, gene knockout studies documented abnormal spleen morphology in the absence of Copine 5 and abnormal cecum, skin, and bone morphology in the absence of Copine 7 [27]. Recent studies indicate that Copines are involved in several pathological mechanisms that are associated with important human diseases. Copines are strongly linked to cancer biology and have been shown to regulate tumor cell proliferation, migration, and invasion and serve as oncomarkers and prognostic markers (reviewed in [28]).

## 2. Structure of Copines

### 2.1. Conserved Molecular Architecture of Copines

**Figure 1:**
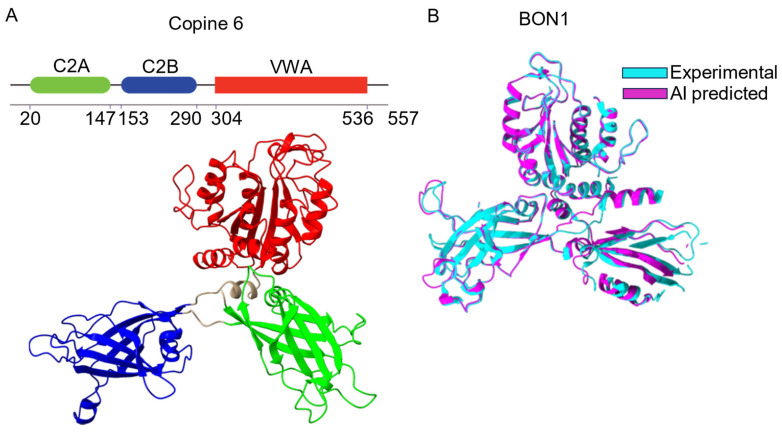
Domain organization of Copines. (**A**) Schematic diagram of human Copine 6 domain structure. C2A (in green), C2B (in blue) and VWA (in red). Domain borders are based on AlphaFold2 structural predictions. Unstructured N- and C-terminal sequences are not shown. (**B**) Comparison of 3D structures of plant Copine protein BON1 (amino acids positions 30–572) determined experimentally (PDB: 6kxk, chain A, in cyan) and predicted by Alphafold2 algorithm (in purple), aligned using ChimeraX software (https://www.cgl.ucsf.edu/chimerax/, accessed on 13 January 2024).

All Copines share a highly conserved and unique architecture containing a pair of C2 domains (C2A and C2B) in the N-terminus followed by a VWA domain interspersed by short linker sequences (Figure 1A) [3]. Mammalian Copines are 59–70 kDa proteins (Table 1). Until recently, the lack of 3D structural information resulted in partial misidentification of Copine domain borders in several biochemical and functional studies, particularly affecting C2 domain boundaries. To date, a 3D structure was determined experimentally only for plant Copine protein BON1 [29]. The arrival of powerful AI-based structure prediction algorithms [30] extended 3D structural information to other members of the Copine family (Table 1).

For BON1, experimental and predicted structures are remarkably similar with minor differences observed in the unstructured loop regions of the protein (Figure 1B). Copine proteins have a butterfly-like structure with “wings” formed by C2A and C2B domains and a “body” made of VWA domain [29]. While the BON1 structure is relatively symmetrical, several mammalian Copines (for example, Copine 6) have a C2A domain aligned closely with the VWA domain and a C2B domain protruding sideways [31] (Figure 1).

**Figure 2:**
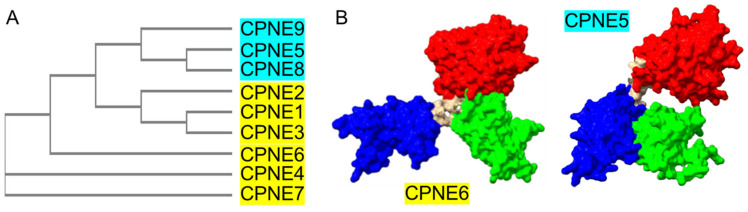
Structural diversification in human Copines. (**A**) Cladogram generated using Clustal Omega multiple sequence alignment of human Copines. (**B**) Two major structural designs in human Copines. Copines 1–4, 6, and 7 are represented by the spacefill model of Copine 6 (AlphaFold: AF-O95741-F1). Copines 5, 8, and 9 are represented by spacefill model of Copine 5 (AlphaFold: AF-Q9HCH3-F1). C2A domains are shown in green, C2B domains are in blue, and VWA domains are in red. Unstructured N- and C-terminal regions are not shown for clarity.

Coincidentally, multiple studies concluded that the C2B domain is critical for calcium-dependent membrane association in various Copines while the C2A domain is dispensable [32,33]. Our phylogenetic analysis of nine human Copines identifies three distinct groups of Copine proteins (Figure 2 and Table 2). 

Copines 1, 2, and 3 are most related to the ancient Copines from lower eukaryotic organisms and are divergent from two other groups, the latter comprising Copines 4, 6, and 7 and Copines 5, 8, and 9, respectively. Our analysis of structural predictions shows the similar organization of Copines 1, 2, 3, 4, 6, and 7 with the butterfly-like shape and a protruding C2B domain. Remarkably, Copines 5, 8, and 9 exhibit a distinct 3D arrangement with their VWA domain positioned further away from the tight bundle formed by C2A and C2B domains (Figure 2). At present, the functional significance of this prospective structural organization is unknown. It is tempting to speculate that Copines 5, 8, and 9 have distinct modes of calcium-activated membrane interaction and membrane aggregation. Several isoforms exist for each of the human Copines genes generated by alternative splicing (Supplementary Table S1). Most of the spliced Copine isoforms contain deletions in the protein sequence or alternative N-terminal sequences. The Copine 7 isoform contains 75 residues—a long loop inserted in the C2A domain. The effect of alternative splicing of Copine genes on their function is currently not clear. 

### 2.2. Tandem C2 Domains and VWA Domain in Copine Proteins

**Figure 3:**
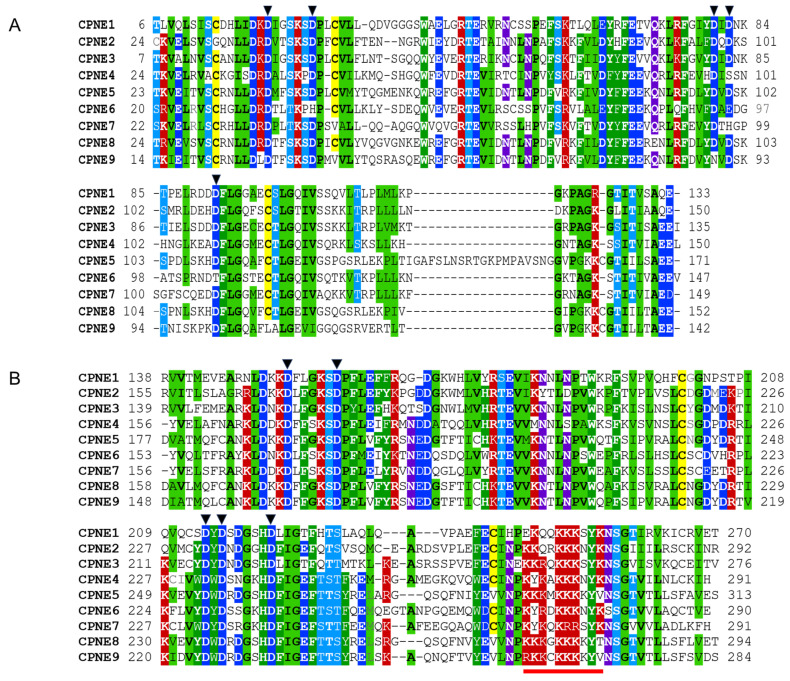
Multiple sequence alignment of human Copine C2 domains. (**A**) C2A domain sequences. (**B**) C2B domain sequences. Structural classes of amino acids are highlighted in color if at least six positions out of nine are conserved in the alignment. Hydrophobic aliphatic residues are in black font highlighted in light green, aromatic residues are in white font and shaded green, cysteines are shaded yellow, basic residues are in white font and shaded red, acidic residues are in white font and shaded dark blue, hydroxyl-containing amino acids are in white font and shaded light blue, amide residues are in white font and shaded purple. Aspartate residues critical for calcium coordination are marked with black triangles.

Tandem C2 domains in Copines belong to the type II class of C2 domains also present in PLC, rabphilin 3A, and other proteins [34]. C2A and C2B domains are connected by short linker sequences of 3–6 amino acids. Both C2 domains in Copines contain highly conserved aspartate residues involved in coordinating two or three calcium ions. Calcium binds to C2 domains with low micromolar affinity (within approximately 1–10 μM range) inducing interaction with negatively charged phospholipids such as phosphatidylserine [35]. The C-terminal part of C2B domains contains an unusual cluster of positively charged lysines and arginines that may potentially serve as a nuclear localization signal. In Copines, C2 domains are thought to mediate transient calcium-dependent association with the plasma membrane. In addition, Copines associate transiently or constitutively with various intracellular organelles, such as Golgi apparatus, clathrin-coated vesicles, and vacuole-like structures [36,37]. The membrane translocation of Copines was observed in multiple systems with the C2B domain playing the dominant role [7,32,33,36,38]. In addition to the ability to bind membranes, C2 domains in Copines are involved in calcium-dependent and calcium-independent protein–protein interactions [31,33,39,40,41]. C2B domains are connected to VWA domains by conserved linkers containing 12–13 amino acids that are likely essential for the correct structural assembly of Copines. Sequences of Human Copine C2A and C2B domains are illustrated in Figure 3. 

**Figure 4:**
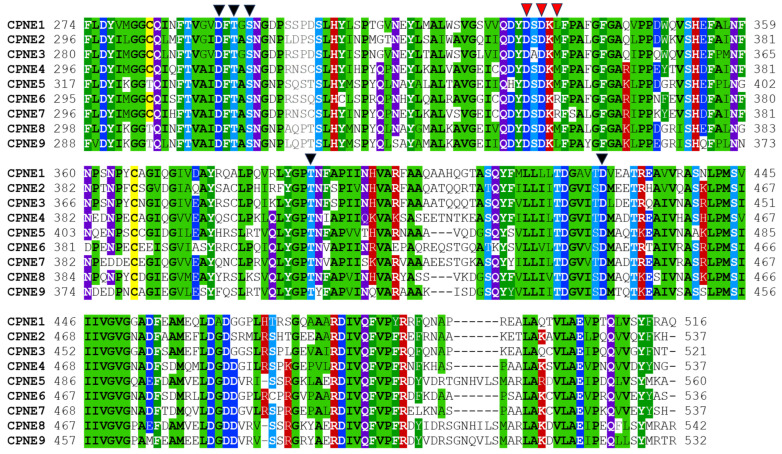
Multiple sequence alignment of human Copine VWA domains. Structural classes of amino acids are highlighted in color if at least six positions out of nine are conserved in the alignment. Hydrophobic aliphatic residues are in black font highlighted in light green, aromatic residues are in white font and shaded green, cysteines are shaded yellow, basic residues are in white font and shaded red, acidic residues are in white font and shaded dark blue, hydroxyl-containing amino acids are in white font and shaded light blue, amide residues are in white font and shaded purple. Amino acids involved in divalent metal coordination at the MIDAS site are marked with black triangles. Residues involved in divalent metal coordination at the additional conserved hypothetical metal binding site are labeled with red triangles.

The VWA domain in Copines binds to multiple proteins in vitro with unclear functional significance [42] and contains a noncanonical metal ion-dependent adhesion site (MIDAS) motif involved in divalent metal binding (Figure 4) [43]. MIDAS motif in mammalian Copines is formed by a discontinuous sequence of five amino acids (D, T, S, T, and D, indicated with black triangles in Figure 4), that are brought in close proximity in the folded protein. MIDAS motif appears to be functional in Copines and binds manganese and possibly magnesium and calcium ions [29,44]. An additional divalent metal binding site was identified in structural studies of plant Copine BON1 that appears to bind calcium in several molecular monomers within the crystal [29]. Calcium-bound monomers had small differences in VWA domain conformation compared to metal-free monomers suggesting that calcium binding to the site may induce structural changes in the Copine molecule. This site is conserved in all mammalian Copines and most identified Copine proteins. The functional significance of the metal binding sites in the Copine VWA domain is presently unclear, however, they are likely involved in regulating effector protein binding similar to integrins and other VWA domain-containing proteins.

## 3. Dynamic Distribution of Copines in Cells and Their Molecular Mechanism of Action

Mammalian Copines 1–3 are ubiquitously expressed, whereas Copines 4–9 have restricted tissue and cell distribution. Copines are soluble cytoplasmic proteins that transiently associate with the plasma membrane or various internal membranes in response to a rise in intracellular calcium concentration. Plant Copines are constitutively associated with membranes through a post-translational modification (myristoylation), which is essential for their function and plant survival [45]. When overexpressed in mammalian HEK293 cells, several Copines showed marked nuclear localization [36,37], however, it is not known whether endogenous Copines are appreciably nucleus-resident proteins. Copines 1 and 3 were detected in circulating exosomes [46,47,48].

The calcium-dependent translocation of Copines to the plasma membrane usually observed with overexpressed proteins and non-physiological calcium levels has led to a widely accepted model stipulating that calcium recruits Copines to the plasma membrane via C2 domains that interact with phospholipids, whilst the VWA domain binds effector proteins. In turn, the effector proteins assemble and concentrate at the plasma membrane to initiate and regulate multiple signaling cascades and downstream processes. Upon the return of calcium to basal levels, the signaling complexes disassemble, terminating the process. The original model of the Copine molecular function proposed in the 1990s faces multiple challenges from the available experimental data. It is difficult to envisage how the VWA domain, a relatively promiscuous protein binding module in cytoplasmic Copines, can confer the high specificity required for tightly controlled signaling events in the cell. Furthermore, several independent screens identified many potential effector proteins that bind C2 domains in Copines. It appears likely that calcium regulation of Copine function might be more complex and involve a conformation switch mechanism found in other calcium-binding proteins such as calmodulin.

## 4. Role of Copines in Mammalian Brain

### 4.1. Copines and Calcium Signaling in Brain

Calcium signaling is critically important for the function of the nervous system. Neurons and glial cells depend on a plethora of calcium-regulated processes, a property shared with all cells in the body. In addition, neurons perform unique functions regulated by calcium that enable information processing in the brain. High temporal and spatial synchronization of action potentials and ensuing neurotransmitter release is of fundamental importance for the productivity and reliability of neural networks [49]. To release neurotransmitters, synaptic vesicles fuse with the plasma membrane in a process mediated by a highly conserved molecular machinery that includes SNARE proteins and accessory proteins [50]. In addition, the synchronization of calcium signal and membrane fusion requires unique calcium-sensing proteins that work alongside the SNARE proteins [51]. The presence of various forms of synaptic neurotransmission, such as synchronous, asynchronous, and spontaneous [52,53], dictates the existence of a network of calcium sensors with distinct properties (calcium affinity, kinetics, cooperativity of calcium binding) that are tailored for a specific function. Several tandem C2 domain proteins including Copine 6, synaptotagmins 1 and 7, and doc2b have been proposed to serve as calcium sensors for various types of neurotransmissions. Calcium signals also initiate many forms of short- and long-lasting changes in synaptic connectivity referred to as synaptic plasticity—a fundamental feature of neural networks required for higher cognitive functions such as learning and memory. Underlying mechanisms for synaptic plasticity are complex and diverse and include functional modifications at the pre- and postsynaptic terminals or structural reconfiguration of synaptic connections [54,55]. One of the best studied sites for synaptic plasticity is the postsynaptic terminal of glutamatergic synapses in mammalian brains where the calcium signal is generated by the activation of the NMDA type of glutamate receptors with multiple downstream targets [56]. Calcium sensors that detect and process the calcium signals are structurally diverse and include both EF-hand proteins (calmodulins) and C2 domain proteins (Copines).

Synaptic transmission and plasticity are not only essential for normal brain function but are also implicated in multiple brain pathologies. These include many devastating neurodegenerative diseases, such as Alzheimer’s disease and Parkinson’s disease, epilepsy, traumatic and stroke-induced brain injuries, and others [57,58]. Copines are associated with synaptic function and brain diseases and have great potential as neuropharmacological targets. A better mechanistic understanding of Copine function in the brain is an essential stepping stone towards this goal.

### 4.2. Spatiotemporal Heterogeneity of Copine Expression in Mammalian Brain

**Figure 5:**
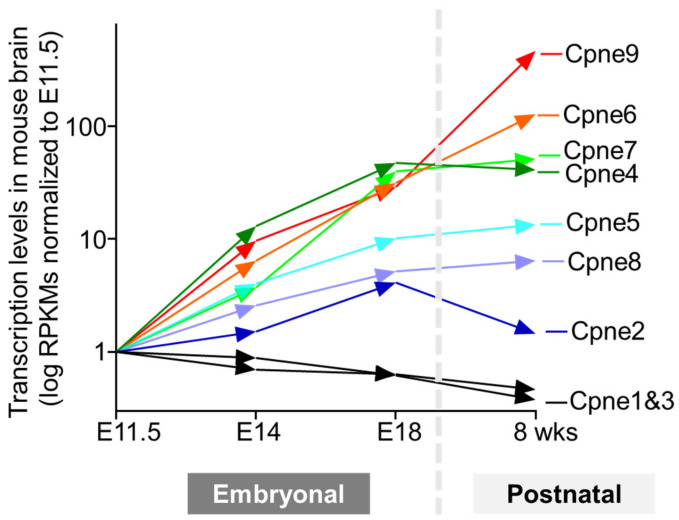
Copine expression in the mouse brain during embryonal and postnatal development. Copine transcription levels as reads per kilobase per million reads mapped (RPKM) sampled at days 11.5, 14, and 18 of embryonal stages and at 8 weeks after birth. The expression data for each Copine were normalized to the earliest time point (mouse embryonal E11.5).

All nine Copine genes are expressed in the mammalian brain according to transcriptomics measurements and many were also detected at the protein level (Human protein atlas. Available online: https://www.proteinatlas.org/search/copine (accessed 5 December 2023)) [59]. Remarkably, Copines display highly varied distribution ranging from the ubiquitous expression at similar levels in all brain regions and cell types to the significant enrichment in specific types of neurons and glial cells. For example, Copine 1 is expressed in all neurons and glial cells at similar levels, while Copines 3 and 4 are highly expressed in Schwann cells and excitatory neurons, respectively. Such variability in Copine expression patterns likely reflects the diverse roles of Copine genes from housekeeping in all cells to specialized functions in distinct cell types. Developmental profiling of Copine expression in mouse brains during embryonal and postnatal stages reveals striking differences for the nine Copine genes (Mouse ENCODE transcriptome data. Available online: https://www.ncbi.nlm.nih.gov/bioproject/PRJNA66167/ (accessed 12 December 2023)) [60], summarized in (Figure 5). 

Based on their expression profiles, Copines can be classified into three categories: (a) gene expression does not change during brain development (Copines 1 and 3); (b) gene expression is modestly increased (<10-fold, Copines 2, 5, and 8); (c) gene expression is upregulated substantially (>10-fold, Copines 4, 6, 7, and 9) reaching approximately 100 to 600-fold increased levels for Copine 6 and 9. The first two weeks of postnatal development in rodents coincide with a large increase in axonal and dendritic projections, their arborization, and synaptogenesis. It seems likely that Copine 6 and 9 might be involved in synaptic function, whereas Copine 1 and 3 are predominantly involved in common cell functions. Transcriptomics measurements of Copine levels in adult rat brains reveal stable expression of all nine genes at 2 weeks (immature animal), six weeks (young adult), 21 weeks (mature adult), and 104 weeks (aged animal), indicating that Copines are required throughout the animal lifespan and exhibit no significant age-dependent expression decline (a rat RNA-Seq transcriptomic BodyMap across eleven organs and four developmental stages. Available online: https://ncbi.nlm.nih.gov/bioproject/PRJNA238328/ (accessed 12 December 2023)) [61]. In retina and retinal ganglion cells (RGC), multiple Copines are differentially expressed at the mRNA and protein levels with the exception of Copines 1, 2, and 7 [37]. Copines 4, 6, 8, and 9 but not Copine 5 show a massive increase in expression at postnatal day 3 compared to embryonal levels at E15. Copines 4, 5, 6, and 9 can be co-expressed in the same neuron, suggesting that different Copines may have distinct properties within the same cell.

### 4.3. Neuronal Copine 6

#### 4.3.1. Dynamic Expression of Copine 6 in Brain

Copine 6 is predominantly expressed in the brain, mainly in the somato-dendritic and synaptic regions of neurons. Copine 6 mRNA is abundant in retinal horizontal and bipolar cells, astrocytes, and inhibitory neurons [33,35,62]. Copine 6 (N-Copine) was the earliest discovered mammalian Copine protein identified in a screen for genes upregulated by neuronal activity [2]. Application of glutamate receptor agonist kainate or high-frequency electrical stimulation of CA1 region in hippocampus induced transient increase in mRNA levels of Copine 6. More recently, a strong increase in Copine expression at mRNA and protein levels was detected in the brains of patients with epilepsy and rats with epilepsy induced by pilocarpine injection [63]. Copine 6 mRNA levels increased 6.6-fold in the neocortex of patients with epilepsy and 2.8-fold and 4-fold in the hippocampus and the cortex of epilepsy-induced rats, respectively. Copine 6 protein levels increased ~4.8-fold in the neocortex of patients with epilepsy and 2-fold and 2.5-fold in the hippocampus and the temporal cortex of epilepsy-induced rats, respectively. Copine 6 levels were also reported to increase substantially during axonal maturation and branching in the mouse lateral olfactory tract axons [64]. There, axons were relatively immature at embryonal E14.5 but underwent rapid maturation and branching at E16.5 and later stages. Copine 6 mRNA levels increased 3-fold by E16.5 and the protein levels increased 3-fold by E16.5 and 20-fold by E18.5. These findings indicate that Copine 6 undergoes a massive increase during synaptogenesis and development of axons and dendrites and even further increases upon neuronal stimulation, suggesting that Copine 6 is involved in synaptic function and plasticity [35].

#### 4.3.2. Copine 6 Role in Neurotransmission

**Figure 6:**
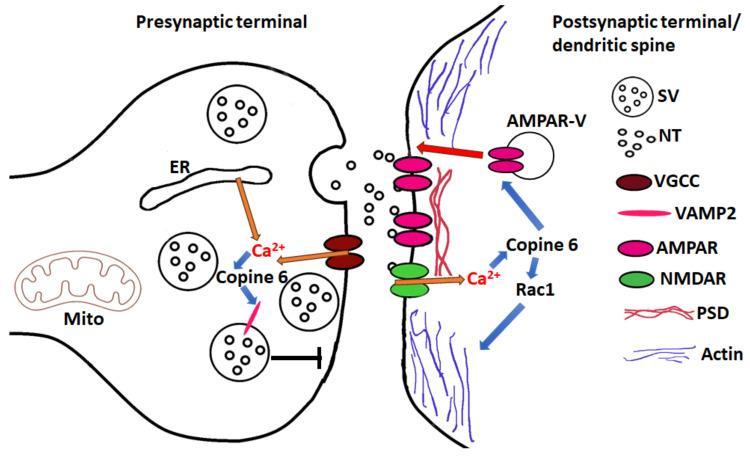
Pre- and postsynaptic functions of neuronal Copine 6. At the presynaptic terminal, Copine 6 binds synaptic vesicle (SV) SNARE protein VAMP2 and downregulates spontaneous neurotransmission. Calcium is released through voltage-gated calcium channels (VGCC) or from internal stores, such as endoplasmic reticulum (ER) or mitochondria (Mito). At the postsynaptic terminal or dendritic spine, Copine 6 recruits Rac1 to the plasma membrane in the presence of calcium and initiates remodeling of actin cytoskeleton followed by changes in spine morphology. Copine 6 also interacts with glutamate receptors, AMPA type (AMPAR), and facilitates AMPAR delivery to the plasma membrane by calcium-regulated exocytosis of AMPAR transport vesicles (AMPAR-V). For both functions, calcium comes through activated glutamate receptors NMDA type (NMDAR). NT denotes ‘neurotransmitter’; PSD—‘postsynaptic density’.

In a search for Copine 6 effector proteins, OS-9 was identified as a calcium-dependent binding partner interacting with the C2B domain of Copine 6 [39]. Notably, a truncated C2B domain was used in these experiments. Later studies revealed that OS-9 is an ER-resident lectin [65] whereas Copine 6 is a soluble cytoplasmic protein. An unbiased screen for calcium-dependent interactors of neuronal SNARE proteins identified Copine 6 binding to v-SNARE protein VAMP2 found on synaptic vesicles and other SNARE proteins [41]. The interaction requires calcium and full-length Copine 6, however, it can be reproduced using “split” Copine 6, a mixture of C2AB and VWA domains. In addition, a C-terminal juxtamembrane linker in VAMP2 is required for binding; truncation of the linker sequence or mutation of vicinal tryptophane residues abolishes the interaction (Figure 6).

Up- or downregulation of Copine 6 in rodent primary hippocampal neurons had no effect on evoked neurotransmission but decreased or increased the frequency but not the amplitude of excitatory or inhibitory miniature potentials correspondingly. The effect of Copine 6 on spontaneous neurotransmission depended on calcium and VAMP2. These observations indicate that Copine 6 is a negative regulator of spontaneous neurotransmission, and the mechanism likely involves the calcium-dependent interaction of Copine 6 with synaptic vesicle SNARE protein VAMP2 (Figure 6). Because Copine proteins are expressed at high levels in the hippocampus, a brain region directly involved in learning and memory, Copines may contribute to the high fidelity of the information processing in the hippocampus required for these advanced brain functions. However, the exact molecular mechanism of spontaneous neurotransmission regulation is currently unknown.

#### 4.3.3. Copine 6 and Synaptic Plasticity

Copine 6 is an attractive candidate for the role in synaptic plasticity because (a) the expression of Copine 6 is regulated by neuronal activity; (b) Copine 6 binds calcium via C2 and VWA domains, initiating translocation of Copine 6 to the plasma membrane or internal membranes and protein–protein interactions; and (c) these properties of Copine 6 are transient and reversible. One of the best-studied sites for synaptic plasticity is the postsynaptic terminal of a glutamatergic synapse in the mammalian brain usually situated on a dendritic spine. Changes in the number or shape of dendritic spines result in long-lasting structural plasticity, whereas modification of the strength in the existing synaptic connections results in more labile functional plasticity. Recent studies implicated Copine 6 in both forms of synaptic plasticity in the postsynaptic terminals of excitatory neurons from rodent brains [31,33,35]. Stimulation of postsynaptic terminals with glycine that activates NMDA receptor-mediated calcium influx and initiates chemically induced long-term potentiation (cLTP) resulted in transient translocation of overexpressed Copine 6 into dendritic spines [33]. Similar effects were observed with endogenous Copine 6 protein in postsynaptic terminals treated with BDNF [35]. Recruitment of Copine 6 to dendritic spines required functional C2B and possibly C2A domains [31]. Activity-induced translocation of Copine 6 coincided with the morphological changes in dendritic spines. Overexpression of Copine 6 resulted in an increase in mushroom-shaped spines and a decrease in filopodia, whereas acute knockdown of Copine 6 had the opposite effect, leading to a decrease in mushroom and stubby-shaped spines and a sharp increase in filopodia. In mice constitutively lacking Copine 6, no significant changes were observed in spine density and morphology, likely due to compensatory mechanisms during mouse development. Importantly, mutant Copine 6 bearing D167N substitution in the C2B domain did not translocate into dendritic spines upon stimulation and also blocked structural plasticity induced by activity establishing a functional link between Copine 6 translocation and structural plasticity. The downregulation of Copine 6 levels in neurons resulted in complex changes in synaptic function. The most consistent change was observed in LTP induced by high-frequency electrical stimulation of acute hippocampal slice cultures. In mice lacking Copine 6 constitutively and in hippocampal slices with acute Copine 6 knockdown, LTP was substantially defective. Remarkably, no major changes in spontaneous neurotransmission were detected in either of the studies. Spine structural remodeling involves changes in actin cytoskeleton dynamics. Copine 6 was shown to bind small GTPase protein Rac1 involved in cytoskeleton organization and dynamics [33]. Binding to Copine 6 activated Rac1 and recruited it to dendritic spines, suggesting that Copine 6 may act as a calcium sensor for NMDA-dependent spine structural plasticity and LTP by regulating actin cytoskeleton via Rac1/LIMK1/Cofilin pathway (Figure 6). Copine 6 was also demonstrated to bind BDNF receptor TrkB and to promote BDNF-TrkB signaling by increasing TrkB surface recycling [35]. In conclusion, Copine 6 emerges as an important regulator of activity-dependent structural plasticity in excitatory neurons. Copine 6 was also implicated in functional synaptic plasticity by a recent study that demonstrated Copine 6 role in activity-induced AMPA receptor exocytosis at the postsynaptic terminal of rodent excitatory hippocampal neurons [31]. Knockdown of Copine 6 reduced the activity-dependent surface delivery of AMPA receptors but had no effect on the basal AMPA receptor surface levels. This effect required the binding of C2 domains in Copine 6 to calcium and to the AMPA receptor GluA1 subunit. The molecular mechanism of how Copine 6 stimulates AMPA receptor exocytosis is presently not clear. Previous studies implicated Synaptotagmins 1 and 7 in calcium-dependent postsynaptic AMPA receptor surface delivery induced by LTP [66]. Remarkably, in neurons lacking Synaptotagmin 1 and 7, endogenous Copine 6 levels were reduced but Copine 6 overexpression restored activity-induced AMPA receptor surface delivery. These findings indicate that Copine 6 is a member of a calcium sensor network involved in functional synaptic plasticity expressed at glutamatergic excitatory synapses.

#### 4.3.4. Copine 6 in Brain Dysfunction

Copine 6 is implicated in several pathological processes affecting brain function. In the brains of epileptic patients, increased expression of Copine 6 may be involved in the pathological mechanism through association with adenylate kinase 5 [67]. In rats fed with a high-fat diet, impaired learning and memory correlate with a decrease in hippocampal expression of Copine 6 providing an intriguing link between metabolism and brain function [68].

### 4.4. Ubiquitously Expressed Copines 1 and 3

Copine 1 is ubiquitously expressed in neurons and glia. Copine 1 is implicated in the early stages of neural development in mice as its levels are transiently upregulated at embryonal E8.5–10.5 during the neural tube closure [69]. Copine 1 is also involved in neuronal differentiation in immortalized rat hippocampal cell line HiB5 and rodent and human neural stem cells [70,71]. Overexpression of Copine 1 induced an increase in neurite outgrowth, expression of neuronal markers, and phosphorylation of PKB/Akt in HiB5 cells. Copine 1 downregulation in HiB5 cells had the opposite effects resulting in a decrease in neurite outgrowth and phosphorylation of PKB/Akt. Similar effects were observed with Copine 1 containing mutations in calcium-binding sites of C2A and C2B domains or with individual mutant C2 domains, suggesting that the role of Copine 1 in neuronal differentiation is calcium-independent and is mediated by PKB/Akt signaling pathway [72]. Notably, in past studies, C2 domain boundaries in recombinant constructs were not determined accurately and no direct measurements of calcium binding to the mutant C2 domains were performed. Several proteins that bind to Copine 1 and that may potentially mediate its effect on neuronal differentiation have been identified [40,73]. These include 14 3 3γ, Jab1, and HAX1, which interact with the C2A domain of Copine 1. Finally, Copine 1 was shown to be involved in the proliferation and neuronal differentiation of human and rodent neural stem cells [71]. Knockdown of Copine 1 in these cells resulted in the downregulation of mTOR signaling, suggesting that Copine 1 regulates the differentiation of neural stem cells by stimulating the PKB/Akt-mTOR pathway. 

Copine 3 is expressed in most tissues, with the highest expression in Schwann cells. Shorter 3′UTR transcripts of Copine 3 were identified in the brains of patients with schizophrenia [74], indicating that dysregulation of alternative splicing in this gene may be clinically relevant for psychiatric disorders. The genome-wide association study (GWAS) detected that higher anxiety correlates with lower working memory for the genotypes associated with reduced expression of Copine 3 [75]. Copine 3 (also other Copines except for Copine 1) was shown to bind the intrinsically disordered N-terminal of prion proteins from Alzheimer’s brains, but not from the normal brains [76]. Overall, these results implicate Copine 3 in several brain disorders with yet unclear molecular mechanisms.

### 4.5. Copines with Restricted Tissue Distribution: Copines 4, 5, 7 and 8

Copine 4 is highly expressed in the prostate and in the brain, where it is almost exclusively found in excitatory neurons. Copine 4 is selectively expressed from the mouse embryonal E13.5 in intrinsic hand and foot innervating motor neurons under the control of Hox genes [77]. Copine 4 is highly expressed in rodent retina, particularly in retinal ganglion cells (RGS). The overexpression of Copine 4 in RGS results in significant morphological changes, such as the induction of large varicosities on the dendrites without any major effect on axons or dendrites possibly by affecting cytoskeleton dynamics [78]. The Copine 4 retinal interactome includes over 200 proteins involved in endolysosomal and autophagosomal pathways, synaptogenesis, and synaptic function. In a search for copy number variations associated with the age at onset of Alzheimer’s disease, two linked CMVs caused a deletion in the Copine 4 gene [79].

Copine 5 is highly expressed in the brain, with high levels found in retinal horizontal cells, oligodendrocyte precursor cells, and excitatory neurons. Copine 5 is upregulated in mice lacking Pbx1 and Pbx3, which serve as Hox co-factors essential for the development and arrangement of spinal motor neurons [80]. Copine 5, and possibly other Copine isoforms, may serve as effector proteins for Pbx-dependent pathways that control neuronal differentiation, synaptic connectivity, and organization. Localization and expression of Copine 5 in developing mouse brains were studied using the antibody generated against the N-terminal region of the protein (amino acids 1–141) [81]. Remarkably, the protein expression was detected in the developing brain but not in the adult brain, which is contrary to the transcriptomics data (Figure 5) and the data on the distribution of Copine 5 in the mouse retina [37]. At present, the reason for these contradictory findings is not clear. One potential explanation might be that the antibody used in [81] did not recognize all Copine 5 isoforms generated by alternative splicing of the Copine gene, known to yield the largest number of Copine isoforms. Copine 5 protein expression was also detected in neural progenitor cells and in the differentiated neurons where it co-localizes with the neural progenitor cell marker Nestin and the neuronal marker Tuj1 correspondingly. Overall, these findings point to the multifaceted involvement of Copine 5 in brain development. In a subsequent study, the same group also generated a constitutive knockout mouse line by deleting the 5′-upstream sequence and exon 1 in the Copine 5 gene [82]. The mice were viable and had no gross abnormalities in overall anatomy. The mice performed normally in locomotor tasks and memory tests but showed decreased anxiety levels in the elevated platform and the elevated plus maze behavioral tests, suggesting that Copine 5 may be involved in regulating anxiety levels. However, it is not clear whether deletion of these sequences that include 32 amino acids at the N-terminus can effectively abrogate all alternatively spliced variants of Copine 5. Finally, single nucleotide polymorphisms (SNPs) in Copine 5 genes were associated with alcohol dependence and obesity, suggesting that Copine 5 may be involved in neuropsychiatric disorders in humans [83].

Copine 7 is expressed in several tissues including the brain, particularly in retinal horizontal cells and bipolar cells, in excitatory and inhibitory neurons. Copine 7 is strongly expressed in the rodent sublaterodorsal nucleus (SLD), a brain area associated with REM sleep regulation [84]. Copine 7 constitutive knockout mice display normal sleep patterns under basal conditions but an increased amount of REM sleep following exposure to novel or stressful conditions and lower theta power density during REM sleep. Selective activation of Copine 7-positive neurons in the SLD using chemogenetics reduced the amount of REM sleep and increased non-REM sleep episodes. These results implicate Copine 7 in REM sleep regulation in rodent brains.

Copine 8 is expressed in many tissues including the brain, where it is highly expressed in excitatory neurons. Copine 8 was identified as a strong candidate gene that regulates prion disease incubation time in mice [85]. In addition, Copine 8 is upregulated in prion-infected mice at the terminal stages of the disease. Together with the previously described observations that Copines may physically interact with the cellular prion protein [76], these results suggest that Copines play an important role in prion disease pathogenesis.

### 4.6. Copines with Unknown Functions in the Brain: Copines 2 and 9

Copine 2 is a ubiquitously expressed protein with high expression in oligodendrocytes, and lower expression in neurons (inhibitory > excitatory). Copine 9 is enriched in the brain with high expression in excitatory neurons.

## 5. Conclusions

Copines are a family of conserved calcium-binding proteins with unique architecture and a multitude of biological functions from protists to humans. In mammals, there are nine Copine genes with distinct patterns of tissue and cell distribution. Copines are made of a tandem of C2 domains, and a VWA domain that bind calcium and are involved in calcium-dependent and calcium-independent protein interactions. In addition, C2 domains in Copines bind phospholipids and mediate transient translocation of Copines to the plasma membrane and internal membranes. Copines play important roles in brain function, ranging from neural development to synaptic function and plasticity, and are implicated in several brain disorders. All Copines are expressed in the brain, but the best-studied isoform is neuronal Copine 6 with a developmentally and activity-regulated expression that is implicated in synaptic function at the pre- and post-synapse. The brain functions of several Copines presently remain unknown.

## Figures and Tables

**Table 1:** Copine proteins and their domains.

Copine Proteins (aa)	Molecular Weight ^1^ (with PTMs), Da	Copine Sequences ID (Name)	Domains and Domain Boundaries ^2^		
			C2A	C2B	vWA
*Human Copines:*					
Copine 1 (1-537)	59,058 (59,378)	Q99829 (CPNE1)	T6-E133	R138-T270	Q283-Q516
Copine 2 (1-548)	61,190 (61,190)	Q96FN4 (CPNE2)	C24-E150	R155-R292	Q305-H537
Copine 3 (1-537)	60,131 (60,850)	O75131 (CPNE3)	T7-I135	R139-V276	Q289-T521
Copine 4 (1-557)	62,395 (62,395)	Q96A23 (CPNE4)	T24-L150	Y156-H291	Q305-G537
Copine 5 (1-593)	65,734 (66,373)	Q9HCH3 (CPNE5)	T23-E171	D177-S313	Q326-A560
Copine 6 (1-557)	61,991 (62,071)	O95741 (CPNE6)	S20-V147	Y153-E290	Q304-S536
Copine 7 (1-633)	70,294 (70,454)	Q9UBL6 (CPNE7)	S22-D149	Y156-H291	Q305-H537
Copine 8 (1-564)	63,108 (63,348)	Q86YQ8 (CPNE8)	T24-E152	D158-T294	Q307-R542
Copine 9 (1-553)	61,864 (62,067)	Q8IYJ1 (CPNE9)	T14-E142	D148-S284	Q297-R532
*Plant Copine:*					
BONZAI 1 (1-578)	63,119 (63,199)	Q941L3 (BON1)	Q49-E188	I194-V326	E339-I570
			S48-E188 ^3^	I194-V326 ^3^	E339-N572 ^3^

^1^ Molecular weight calculated based on the protein sequence alone prior to post-translational modifications (PTMs) and separately, including PTMs (based on the relevant UniProt entries, from https://www.uniprot.org, accessed on 14 February 2024); ^2^ Boundaries of C2A, C2B, and VWA domains in human Copines 1–9 and plant Copine BON1 predicted by Alphafold2 algorithm unless specified otherwise.; ^3^ Boundaries of C2A, C2B, and vWA domains in plant Copine BON1 deduced from X-ray structure (PDB: 6KXK).

**Table 2:** Human Copines—protein sequence comparisons.

	Identity ^1^	Copine 1	Copine 2	Copine 3	Copine 4	Copine 5	Copine 6	Copine 7	Copine 8	Copine 9
Similarity ^1^										
Copine 1			59%	66%	52%	49%	51%	54%	52%	48%
Copine 2		75%		65%	54%	52%	51%	57%	52%	49%
Copine 3		80%	80%		57%	53%	55%	58%	53%	50%
Copine 4		69%	71%	73%		50%	63%	74%	53%	49%
Copine 5		66%	68%	67%	65%		48%	45%	80%	77%
Copine 6		67%	67%	70%	79%	62%		71%	50%	47%
Copine 7		70%	75%	76%	88%	59%	86%		53%	51%
Copine 8		69%	69%	71%	68%	89%	67%	67%		79%
Copine 9		68%	69%	68%	66%	87%	63%	66%	90%	

^1^ BLASTP pairwise alignment of human Copine protein sequences.

## Supplementary Materials

The following supporting information can be downloaded at: https://www.mdpi.com/article/10.3390/biom14030255/s1, Table S1: Human Copine proteins and their isoforms.
