# Supplementary material for: Copines, a Family of Calcium Sensor Proteins and Their Role in Brain Function
Source: Biomolecules. 2024 Feb 21;14(3):255. doi: 10.3390/biom14030255 (PMC10968396; doi:10.3390/biom14030255)
Supplement: Supplementary file 1 [file biomolecules-14-00255-s001.zip › biomolecules-2847673-supplementary.pdf]

biomolecules

Copines, a family of calcium sensor proteins and their role in brain function

Mikhail Khvotchev [1,*] and Mikhail Soloviev [2,*]

[1] Department of Biochemistry, Center for Neuroscience, Faculty of Science, Mahidol University, Bangkok 10400, Thailand
[2] Department of Biological Sciences, Royal Holloway University of London, Egham, Surrey TW20 0EX, UK
* Correspondence: mikhail.khv@mahidol.edu (M.K.); mikhail.soloviev@rhul.ac.uk (M.S.)

Supplementary Table S1. Human Copine proteins and their isoforms.

Canonical Copine proteins (aa)	Canonical Copine sequences ID (name)	Copine isoforms and potential isoforms [1]	
		Isoform sequences ID	Length (aa)
Copine 1 (1-537)	**Q99829** (CPNE1)	**Computationally mapped Copine 1 isoforms**	
		B0QZ18	542
		F2Z2V0	533
		A6PVH9	481
		E7ENH5	427
		Q5JX45	386
		Q5JX52	305
		Q5JX44	304
		Q5JX60	231
		Q5JX61	213
		Q5JX59	209
		H0Y524	208
		Q5JX58	201
		Q5JX56	174
		Q5JX55	152
		E7EV27	138
		Q5JX57	137
		Q5JX54	136
		Q5JX53	66
Copine 2 (1-548)	**Q96FN4-1** (CPNE2)	**Copine 2 isoform**	
		Q96FN4-2	446
		**Computationally mapped Copine 2 isoforms**	
		H3BUC8	281
		H3BPR8	121
		H3BQQ3	81
Copine 3 (1-537)	**O75131** (CPNE3)	**Computationally mapped Copine 3 isoforms**	
		H0YB26	262
		A0A087WYQ3	234
		E5RHZ0	153
		A0A087WXR6	139
		A0A087WUS8	135
		E5RFT7	79
		A0A0G2JMP5	8

Copine 4 (1-557)	**Q96A23-1** (CPNE4)	**Copine 4 isoform**	
		Q96A23-2	575
		**Computationally mapped Copine 4 isoforms**	
		D6RCT2	169
		D6RI99	82
		D6RFY4	73
Copine 5 (1-593)	**Q9HCH3-1** (CPNE5)	**Copine 5 isoform**	
		Q9HCH3-2	301
		**Computationally mapped Copine 5 isoforms**	
		A0A0J9YWA1	610
		A0A0J9YWU8	226
Copine 6 (1-557)	**O95741-1** (CPNE6)	**Copine 6 isoform**	
		O95741-2	612
		**Computationally mapped Copine 6 isoforms**	
		H0YLV2	226
		H0YKP1	159
		H0YLM2	158
		H0YKJ0	153
		H0YNM7	149
		H0YNX4	141
		H0YNP2	131
		H0YK44	110
		H0YM67	105
		H0YNV6	65
		H0YNV2	52
		H0YLS9	26
Copine 7 (1-633)	**Q9UBL6-1** (CPNE7)	**Copine 7 isoform**	
		Q9UBL6-2	558
		**Computationally mapped Copine 7 isoforms**	
		H0YEH8	173
		E9PJ31	120
		H3BP03	98
		H0YE30	92
Copine 8 (1-564)	**Q86YQ8-1** (CPNE8)	**Copine 8 isoform**	
		Q86YQ8-2	233
		**Computationally mapped Copine 8 isoforms**	
		E7ENV7	552
		F8VZB5	12
Copine 9 (1-553)	**Q8IYJ1-1** (CPNE9)	**Copine 9 isoform**	
		Q8IYJ1-2	503
		**Computationally mapped Copine 9 isoform**	
		H7BXI0	196

[1] Copine proteins isoforms and potential isoform sequences are based on the UniProt entries for the relevant canonical Copines listed in the Table (from https://www.uniprot.org. Accessed 14/02/2024).
